# Perovskite oxides as transparent semiconductors: a review

**DOI:** 10.1186/s40580-020-00242-7

**Published:** 2020-10-02

**Authors:** Haiying He, Zhihao Yang, Yonghang Xu, Andrew T. Smith, Guangguang Yang, Luyi Sun

**Affiliations:** 1 School of Materials Science and Hydrogen Energy, Foshan University, Foshan, 528000 China; 2 Polymer Program, Institute of Materials Science, University of Connecticut, Storrs, CT 06269 USA; 3 Department of Chemical & Biomolecular Engineering, University of Connecticut, Storrs, CT 06269 USA; 4 School of Electronic Information Engineering, Foshan University, Foshan, 528000 China

**Keywords:** Perovskite oxide, Transparent conducting oxide, SrVO_3_, BaSnO_3_, Semiconductor

## Abstract

Traditional transparent conducting oxides (TCOs) have been widely used for various optoelectronic applications, but have the trade-off between conductivity and transmittance. Recently, perovskite oxides, with structural and chemical stability, have exhibited excellent physical properties as new TCOs. We focus on SrVO_3_-based perovskites with a high carrier concentration and BaSnO_3_-based perovskites with a high mobility for n-type TCOs. In addition, p-type perovskites are discussed, which can serve as potential future options to couple with n-type perovskites to design full perovskite based devices.

## Introduction

Transparent conducting oxides (TCOs) are a group of unique materials for optoelectronic applications, including displays, solar cells, and light-emitting diodes [1–4]. High transmittance (≥ 80%) in the visible spectrum, high conductivity, and high carrier mobility are required for transparent electronics. Various metal oxide materials, such as Sn-doped In_2_O_3_ (ITO) [5–7], F-doped SnO_2_ (FTO) [8, 9], Sb-doped SnO_2_ (ATO) [10, 11], and Al-doped ZnO (AZO) [12–15], have been investigated. Researchers have been developing these oxides to exhibit high performance by focusing on transmittance in the visible range, electrical conductivity at room temperature, stability in the working environment, high carrier mobility, and controlling conductive type.

Electrical conductivity is determined by **σ = enμ = e**^**2**^τ (n/m^*^), where **τ, n,** and m^*^ are scattering time, carrier concentration, and electron effective mass, respectively. It is clear that the value of n/m* is the key to achieve high performance of TCOs provided that the scattering time is maximized [16]. Typical post-transition metal cations such as In^3+^ [7, 17], Zn^2+^ [18], and Sn^4+^ [19] have largely spread vacant s-orbitals to form the conduction band minimum (CBM) of TCOs, along with small effective masses. Among these materials, ITO has been widely used in industry thanks to its highest conductivity (~ 10^4^ S·cm^−1^), along with an electron carrier concentration of the order of 10^21^ cm^−3^ [5, 20]. However, the carrier concentration is limited and the total scattering time of the carriers could be reduced due to the solubility limitation of the dopants as well as self-compensation [21–23]. Additionally, it is well-known that the markets for transparent devices are large but the critical raw material, Indium (In), is scarce on earth, which has led to high costs [24]. Substitution with ZnO- and SnO_2_-based binary compounds has found success with optimized electrical performance, but this strongly depends on approaches of deposition and growing conditions [25]. It should be noted that it is difficult to deposit high performance impurity-doped metal oxide thin films. The key factors are to improve crystallinity by deposition methods and control an oxidizing atmosphere [25–27]. It was reported that AZO thin films deposited by pulsed laser deposition (PLD) showed an encouraging conductivity of the order of 10^4^ S·cm^−1^ [15].

Carrier mobility (**μ**), defined as **μ =** eτ/m^*^, is another important factor for the high performance of transparent oxides. Conduction bands derived from widely spread metal s orbitals result in n-type conductivity and thus excellent electron carrier mobility, whose values range from 10 to 100 cm^2^·V^−1^·s^−1^. However, the applications of transparent oxides can hardly be further developed due to the lack of p-type counterparts for p-n heterojunctions [20, 28, 29] or other p-type devices like photovoltaics [3]. So far no efficient method has been created to introduce shallow acceptors and small hole effective mass due to strongly localized O 2p orbital derived from valence bands of metal oxides, though huge efforts have been made [30]. The carrier mobility of p-type TCOs cannot compare with those of n-type, even though high mobility values have been achieved from Cu-based materials [31, 32] at the sacrifice of hole carrier concentration.

ABO_3_-based perovskite oxides are common in inorganic compounds. The ideal cubic perovskite structure (*Pm*-*3* *m*) is composed of an A-site cation (an alkaline-earth, a rare-earth or an alkali element), B-site cation (a transition or post-transition metal element from periods 4, 5, or 6 in the periodic table), and oxygen anions. B-site cation sits at the center of BO_6_ octahedron [65]. It should be noted that distortions are caused by cations of different radii, which results in octahedral tilting. The structure of perovskite oxides can remain stable even if A- or B-site cations are replaced by a large amount of other elements [33, 34]. Materials with a perovskite structure have drawn high attention for extensive research on a variety of physical properties, including superconductivity [35], metal–insulator transition [36], photovoltaic effect [37], transparent conductivity [38–43], magnetism [44], and ferroelectricity [37, 45]. In particular, using novel perovskite oxides to replace traditional binary TCO materials has become popular. Recently, Zhang et al. [16] reported that correlated metal compounds (e.g., SrVO_3_ and CaVO_3_), as transparent conductors, had an excellent carrier concentration (> 2.2 × 10^22^ cm^−3^), an order of magnitude greater than that of ITO (~ 10^21^ cm^−3^). Alkaline earth stannates (ASnO_3_, A = Ca, Sr, Ba) have a wide bandgap (~ 3.0 eV), exhibiting highly transparent visible photovoltaic effective range and excellent electrical properties [46–50], i.e., a mobility up to 320 cm^2^·V^−1^·s^−1^ at room temperature [51, 52]. In contrary to stoichiometric perovskite oxides that are insulating, oxygen-deficient structures transform to semiconductor or conductor, i.e., oxygen vacancy also plays a role in electrical properties [53, 54]. Early research results have revealed high thermal stability of oxygen in perovskite materials [55, 56], thus avoiding the degradation in the oxide-based electronic devices.

On the other hand, it remains a challenge to achieve p-type conductivity by binary post-transition metal oxide because of strongly localized O 2p orbitals, resulting in high formation energy of acceptor and large hole carrier masses. Fortunately, A or B sites can easily be replaced by other elements in perovskite oxides, thanks to the high chemical and structural stability. Stoichiometric strontium titanate (SrTiO_3_, STO) is a group of highly transparent insulators (Eg ~ 3.2 eV) that can be easily doped by Nb [57], La [58], or Sb [59] for n-type STO, or doped by Sc [60], and In [61] for p-type ones. Like “chemical modulation of the valence band” (CMVB), introduced by Hosono et al., partial replacement of Al for Sn in cubic perovskite-structures, SrSnO_3_, can decrease the localization and generate holes in the valence [62]. Perovskite compounds such as La_1-x_AlSnO_3_ [62], La_1-x_Sr_x_CrO_3_ [42, 63], La_2/3_Sr_1/3_VO_3_ [33], SrTiO_3_:Rh [64], etc., can be p-type TCO candidates, which demonstrate excellent electrical and optical properties. The new perovskite oxides not only balance the trade-off between conductivity and transmittance, but also show p-type characteristics. Development of p-type perovskite oxides are critical, along with n-type perovskites, to make full perovskite devices.

In this article, we review the novel TCO materials based on perovskite oxides. Section 2 discusses the crystal structure, electronic properties, fabrication methods of the SrVO_3_- and BaSnO_3_-based perovskites. SrVO_3_ films inherit a high conductivity thanks to the high carrier concentration, even though they have a high effective mass. BaSnO_3_-based perovskites have a high mobility. Section 3 describes the properties and impurity doping of p-type perovskite oxides. Due to their high chemical stability, perovskite oxides can be doped with A- or B-site.

## n-Type perovskites

### SrVO_3_

**Fig. 1:**
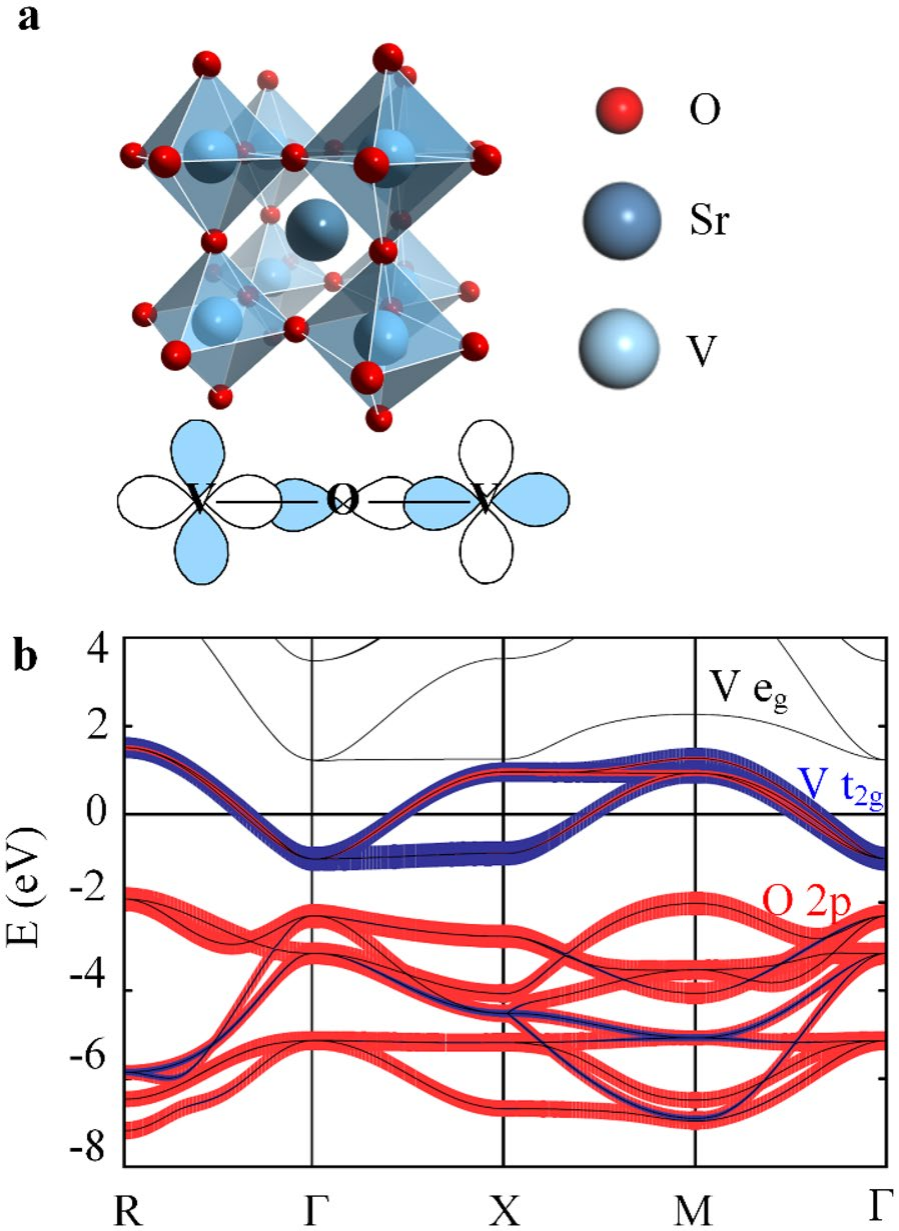
**a** Cubic perovskite structure of SrVO_3_ (a = 3.842 Å). **b** Band structure of SrVO_3_ in the vicinity of the Fermi level E_F_ [68]

Alkaline earth metal vanadium oxides with the AVO_3_ (A = Ca, Sr, Ba) composition have a V^4+^ 3d^1^ electronic configuration with a single electron in the partially filled t_2g_ band. The first-principle calculations showed three t_2g_ bands at the Fermi energy between -1.5 eV and 1.5 eV [66]. In this article, strontium vanadium oxide (SrVO_3_, SVO), which has aroused widespread interest, is selected to illustrate that strongly correlated metal oxides are promising transparent conductive materials. SVO possesses cubic symmetry (a = 3.842 Å) with one V atom surrounded by six O atoms to form an octahedral configuration (Fig. 1a). The valence band maximum (VBM) is composed of O 2p orbitals, and the conduction band minimum (CBM) is composed of V 3d orbitals that can be split into e._g._ and t_2g_ states (Fig. 1b). The width of 3d bands is narrow enough (W ≈ 2-3 eV) so that the local Coulomb interaction between the electrons is particularly strong, causing electronic correlations [67].

SVO is an important strongly correlated metal oxide that has been extensively investigated in both theoretical [68–70] and experimental researches [71–73]. Recently, SVO has been reported as a transparent conductor because of its high electrical properties. Contrary to heavy doping to optimize physical properties in wide-bandgap semiconductors, Zhang et al. [16] demonstrated that novel materials with strong electron–electron interactions had a higher carrier concentration in comparison with that of ITO. This kind of materials inherit a high conductivity (~ 10^4^ S·cm^−1^) due to the higher carrier concentration (~ 10^22^ cm^−3^) although they have a high effective mass. In addition, SVO films show optical transmittance in the visible range due to lower plasma frequency (ω_p_* = 1.33 eV), allowing them to be thinner, to increase optical transmittance in the visible spectrum range without suffering from surface scattering because of the much smaller electron mean free path (EMFP = *ħ*(3π^2^)^1/3^(τ/n^2/3^) (n/m^*^)) in correlated metal oxides (SrVO_3_, EMFP= 5.6 nm and CaVO_3_, EMFP= 3.9 nm) [16] than other conductors, like Ag (EMFP= 52 nm) and Au (EMFP= 50 nm) [74, 75].

However, an inevitable problem is that the carrier mobility and transmittance of SVO is lower than ITO. The factors those affect the physical properties of the SVO thin films, including: (1) Film crystallinity. The SVO thin films with high performance are all crystal phase deposited on lattice-matching substrates, such as (LaAlO_3_)_0.3_(Sr_2_AlTaO_6_)_0.7_ (LSAT), SrTiO_3_ (STO), NdGaO_3_ (NGO), etc., as shown in Table 1, instead of on an amorphous substrate (glass or PET) or other substrates with a different crystal structure (Si, GaN, or Al_2_O_3_). Note that the properties of the films are not directly affected by the lattice mismatch, but mainly determined by lattice misfit between the SVO film (a(SVO)) and the substrate (a_s_), *f *= [a_s_-a(SVO)]/a_s_. The *f* values between SVO bulk and LSAT (3.868 Å), STO (3.905 Å), and NGO (3.863 Å) substrates are +0.67%, +1.61%, and +0.54%, respectively. Under the same growth conditions, the carrier concentration (or mobility) of the SVO films on LSAT and NGO are close, with values of 2.14 × 10^22^ cm^−3^ (2.9 cm^2^·V^−1^·s^−1^) and 2.2 × 10^22^ cm^−3^ (3.3 cm^2^·V^−1^·s^−1^), respectively [76]. However, the resistivity of the SVO film grown on an LSAT substrate (70 μΩ·cm) is lower than that on a STO substrate (90 μΩ·cm) [77]. (2) Growing methods. To grow high quality thin films, various growing methods have been developed to deposit SVO films to achieve high performance, including pulsed laser deposition (PLD) [76–80], molecular beam epitaxy (MBE) [81], hybrid MBE [43, 82, 83], and radio frequency sputtering deposition (RF sputtering) [84]. These results show that preparation method has a great influence on the properties of the prepared films. This might be caused by the defects generated in the preparation process. For example, good stoichiometric SrVO_3_ can be controlled by hybrid MBE, which can reduce the number of defects of perovskite-based oxide films [82, 83]. The resistivity (28 μΩ·cm [16]) of the SVO films grown by MBE measured at room temperature is lower than those grown by other deposition approaches like PLD (38 [79] and 120 μΩ·cm [80]).

**Table 1:** Summary of the electrical properties of SrVO_3_ thin films compared to ITO

Substrate	Deposit methods	Mobility cm^2^·V^−1^·s^−1^	Carrier concentration cm^−3^ (× 10^22^)	Resistivity Ω·cm (× 10^−5^)	Ref.
Sn:In_2_O_3_ (ITO) On glass substrate	Reactive e‐beam evaporation	~30	0.08	~0.2	[6]
SrTiO_3_ (STO)	PLD	1.35	2.48	0.187	[78]
(LaAlO_3_)_0.3_(Sr_2_AlTaO_6_)_0.7_ (LSAT)	MBE	~10^a^	2.26	2.8	[16]
(LaAlO_3_)_0.3_(Sr_2_AlTaO_6_)_0.7_ (LSAT)	Hybrid-MBE	~9^a^	–	3	[43]
NdGaO_3_ (NGO)	PLD	8.3	2.4	3	[76]
(LaAlO_3_)_0.3_(Sr_2_AlTaO_6_)_0.7_ (LSAT)	PLD	~7^a^	2.6	3.8	[76]
(LaAlO_3_)_0.3_(Sr_2_AlTaO_6_)_0.7_ (LSAT)	PLD	3.05	2.18	0.12 ~ 0.19	[80]
(LaAlO_3_)_0.3_(Sr_2_AlTaO_6_)_0.7_ (LSAT)	MBE	8.7	2.3	3.2	[81]
(LaAlO_3_)_0.3_(Sr_2_AlTaO_6_)_0.7_ (LSAT)	Sputtering	1.82	1.57	0.2	[84]

^a^Values estimated graphically

An open question still facing SVO materials is if it will be one of the candidates to replace ITO in many optoelectronic devices. Except for high optical transmittance and high electrical properties, large area deposition, uniformity, and integration of oxide materials are essential. Amorphous materials have obvious advantages in the deposition of large area homogeneous films thanks to their anisotropy properties. Boileau et al. [80] investigated the optical and electrical properties of SVO films without long-range crystalline order. The resistivity and transmittance were found to depend on crystalline state. In the crystalline state, the transmittance reached ca. 80% for a 520 nm film and the resistivity was measured to be 1.2 ~ 1.9 × 10^−4^ Ω·cm, while the electrical properties deceased sharply with a decreasing crystallinity and the resistivity increased by orders of magnitude. The crystallinity of SVO significantly affects the properties of the films, limiting their potential as an alternative to ITO. Besides, the mobility of carriers is low due to the high effective mass induced by strong electron–electron interaction.

Furthermore, the performance of SVO thin films is sensitive to oxygen pressure [77, 79, 85]. In general, SVO is grown under low oxygen pressure, which is different from other strongly correlated perovskite materials, such as LaNiO_3_ and SrRuO_3_ grown under high oxygen pressure conditions [86–88]. At a high oxygen pressure, V^4+^ can be easily oxidized into V^5+^, indicating that optimization of oxygen pressure has a positive effecnt on film properties. It should be noted that the properties of the films prepared in vacuum are not optimal [77, 79]. Wang et al. achieved the highest conductivity at room temperature with an oxygen pressure of 1 × 10^−5^ mbar [77]. The effect of oxygen pressure on the physical properties of thin films is still not completely clear and requires further investigation.

### BaSnO_3_

#### Theoretical calculation

**Fig. 2:**
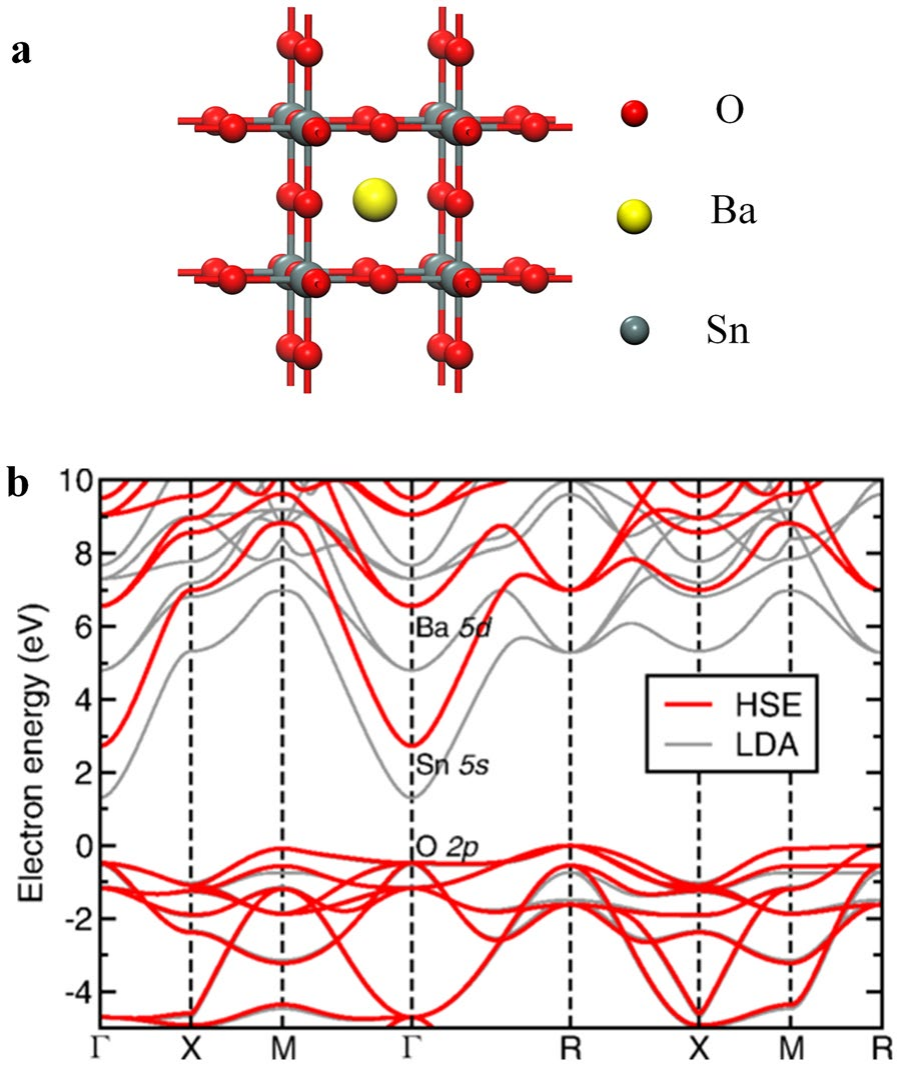
** a** Cubic perovskite structure of BaSnO_3_ (a = 4.115 Å). **b** Band structure of BaSnO_3_ based on the first-principle calculations [102]

The limitation of SVO films for wide applications is their low carrier mobility, which is just on the order of 10 cm^2^·V^−1^·s^−1^. It is difficulty to enhance the mobility because of high effective mass and anisotropic electron carrier transport path. In contrast to strongly correlated SVO, alkaline earth stannates (ASnO_3_, A = Ba, Sr, and Ca) have been reported to have a high mobility, particularly barium stannate (BaSnO_3_). Unlike V 3d, Sn 5 s orbitals are isotropic and can still overlap to a large extent even in the amorphous state, without affecting the conductivity. Metal ions with (n−1)d^10^ns^0^ electronic configuration play a dominant role in high conductivity and high mobility of amorphous semiconductor materials [89, 90]. According to the literature, BaSnO_3_ (belonging to the perovskite family), shows a wide band gap (E_g_ > 3.1 eV) [91], a high thermal stability [55], and a high carrier mobility up to 320 cm^2^·V^−1^·s^−1^ in single crystals at room temperature [51] and 70 cm^2^·V^−1^·s^−1^ in epitaxial films [52], respectively. Caused by the high mobility, BaSnO_3_-based materials have been used as a semiconductor layer in oxide thin film transistors (TFTs) [92–95] and other photoelectronic devices [96–98]. It should be mentioned that the mobility of BaSnO_3_ is much higher than that of SrVO_3_. In contrast to CBM derived mainly from the localized V 3d states, the first-principle calculations showed that CBM is dominated by dispersive Sn 5 s states (Fig. 2), leading to a smaller carrier effective mass [48, 99]. This is similar to other cations with a (n−1)d^10^ns^0^ electron configuration, such as In^3+^, Zn^2+^, and Ga^3+^. Most effective masses of BaSnO_3_ from the first-principle calculations are between 0.2 and 0.5m_0_ [38, 48, 96, 100, 101]. They are apparently smaller than titanium- or vanadium-based perovskites, e.g., BaTiO_3_ (5.3m_0_) [103], and SrVO_3_ (3.0m_0_) [76], but close to traditional semiconductors, such as Si (0.27m_0_) [104], In_2_O_3_ (0.35m_0_) [25], and SnO_2_ (0.25m_0_) [105]. The smallest value below 0.03m_0_ was reported by Moreira et al. with first-principle local density approximation (LDA) and generalized gradient approximations (GGA) calculations [106]. The small effective mass has been found in tin-based perovskites [48], including SrSnO_3_ (0.23m_0_), ZnSnO_3_ (0.17m_0_), and CdSnO_3_ (0.23m_0_). This indicates that cation Sn on B-site is critical to high mobility.

#### Crystalline BaSnO_3_

High mobility has not been measured for stoichiometric BaSnO_3_ and it is known that it is not caused by intrinsic defects, but obtained from La-doped BaSnO_3_. Negative defect formation energy indicates a high concentration of substituting La for Ba (La_Ba_) in BaSnO_3_ [107], while the charge-state transition energy level, E[La_Ba_ (+/0)], with respect to CBM is just 0.046 eV, indicating La_Ba_ to be a shallow donor, which contributes one electron easily by the ionization of La atoms [48]. To be a good candidate for TCOs, the solubility of dopants should be high and the donor level should be shallow. High carrier mobility of Ba_1-x_La_x_SnO_3_ (x = 0 to 0.04) crystals were reported by Luo et al., when x = 0.02 the highest mobility was about 103 cm^2^·V^−1^·s^−1^ corresponding to a carrier concentration of ~ 8-10 × 10^19^ cm^−3^ [108]. Kim et al. [51] enhanced the carrier mobility of (Ba, La)SnO_3_ single crystals to the highest value so far of 320 cm^2^·V^−1^·s^−1^. In the crystal structure of BaSnO_3_, tin atom is at the center of the octahedra (SnO_6_) and the band angle of Sn–O-Sn is ~ 180^o^. When Ba is partially replaced by La, the defects (La_Ba_) are located away from SnO_6_ octahedra, causing a low disorder effect. In addition, at a doping concentration of 8 × 10^19^ cm^−3^, the mobility showed the highest value [51]. For single crystals, the mobility decreases with an increasing carrier concentration due to scattering. Therefore, a suitable doping level can reduce the dopant scattering in comparison with high carrier concentrations (> 10^20^ cm^−3^). Phonon scattering also has an effect on carrier mobility, but such effect is less significant than that of dopant scattering.

#### Epitaxy-grown BaSnO_3_ films

**Fig. 3:**
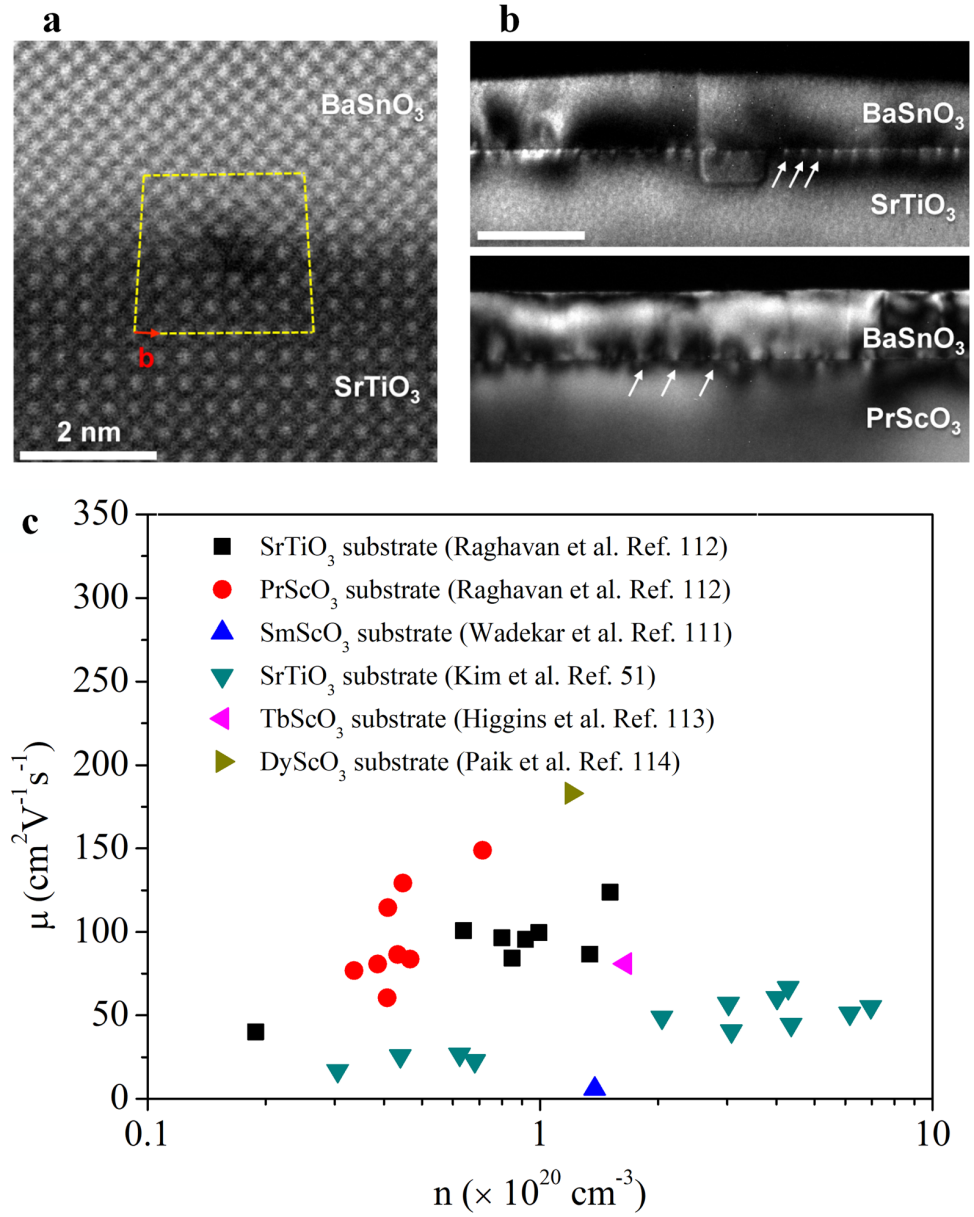
**a** High-angle annular dark-field (HAADF)-STEM image of BaSnO_3_/SrTiO_3_ interface [112]. **b** Cross section dark-field TEM images of BaSnO_3_ on SrTiO_3_ (top) and PrScO_3_ substrates (bottom) [112]. **c** Summary of the mobility of BaSnO_3_ epitaxial films grown on SrTiO_3_ [51, 112], SmScO_3_ [111], PrScO_3_ [112], TbScO_3_ [113], and DyScO_3_ [114] substrates

Compared to single crystals, the mobility value of La-doped BaSnO_3_ epitaxial films is much lower due to grain boundaries and dislocations caused by lattice mismatch between (Ba, La)SnO_3_ and the substrate [109]. To enhance the film mobility, researchers managed to grow La-doped BaSnO_3_ epitaxial films on other cubic perovskites, such as SrTiO_3_(001) [51, 52, 110], SmScO_3_(110) [111], PrScO_3_(110) [112], TbScO_3_(110) [113], and DyScO_3_(001) [114]. The electron mobility was enhanced from 5.8 to 183 cm^2^·V^−1^·s^−1^ at room temperature, as shown in Fig. 3. Furthermore, BaSnO_3_ epitaxial films were grown on BaSnO_3_ single crystal substrate with a mobility of 102 cm^2^·V^−1^·s^−1^ [115]. The mobility can be increased from ~ 37 cm^2^·V^−1^·s^−1^ (without buffer) to ~ 56 cm^2^·V^−1^·s^−1^ at room temperature when a bilayer of BaSnO_3_/(Sr,Ba)SnO_3_ was inserted as a buffer layer to reduce dislocation scattering [116]. Wang et al. [117] reported that La-doped BaSnO_3_ films using SrTiO_3_ buffer on Si(001) substrate showed a mobility of 128 cm^2^·V^−1^·s^−1^ at room temperature. This result is remarkable, but the fabrication cost has been significantly increased. To address this issue, Sanchela et al. [118] developed to grow La-doped BaSnO_3_ films under a highly oxidative ozone (O_3_) atmosphere, achieving a balance between mobility (115 cm^2^·V^−1^·s^−1^) and processing cost.

#### Oxygen vacancy

The intrinsic point defect, oxygen vacancy (V_O_), plays a crucial role in various oxide semiconductors, including ZnO, SnO_2_, and In_2_O_3_. The first-principle calculated results show that the formation energy of V_O_ has a negative value in perovskite stannates [107], indicating defects will form spontaneously along with grown films. BaSnO_3_ films with a high oxygen vacancy concentration were fabricated at low oxygen pressures [54, 119] and reducing conditions (Ar containing 5% H_2_ gas) [120]. Oxygen vacancies are donor defects which could contribute twice as many electron carriers per single-unit defect. Experimentally, high n-type carrier concentrations have been reported under O-poor growth conditions [54, 121, 122]. Son and coworker [123] reported that the mobility of La-doped BaSnO_3_ film was 78 cm^2^·V^−1^·s^−1^ at room temperature by high-temperature annealing under a N_2_ environment to generate V_O_. Later, the same group enhanced the mobility up to 122 cm^2^·V^−1^·s^−1^ by controlling the oxygen pressure using H_2_ environment annealing [120]. In addition, it is known that oxygen vacancies could be generated by a vacuum annealing process in undoped BaSnO_3_ films [124, 125]. Vacuum annealing methods have been reported to treat La-doped BaSnO_3_ films on MgO substrates with a carrier mobility of 101.6 cm^2^·V^−1^·s^−1^. The value was larger than that of the film deposited without annealing (~ 74 cm^2^·V^−1^·s^−1^) [126]. Therefore, it is important to investigate the effect of oxygen vacancies on physical properties of BaSnO_3_ films.

## p-Type perovskites

Conventional TCOs, including ZnO, SnO_2_, and In_2_O_3_, typically exhibit n-type electronic conductivity, and are widely used in various fields. However, the lack of high performance p-type TCOs has limited their applications [127, 128]. In general, p-type TCOs were synthesized by acceptor doping to create holes in the valence band, but the hole concentration is usually not high enough due to recombination and strong localization [129, 130]. The conductivity and hole concentration suffer from doping bottlenecks, intrinsic defects, and the solubility limit of the acceptors. Although previous studies explored some approaches to change n-type TCOs to p-type ones, the hole carrier mobility is usually an order of magnitude lower than that of electron carrier. Moreover, Cu^+^-based oxides (CnMO_2_, M = B, Al, Ga, In, Sc) with d^10^ configuration were used for intrinsically p-type doped materials [131]. Layered oxychalcogenides [132] and d^6^ spinel oxides [133] were reported to achieve p-type conductivity.

**Fig. 4:**
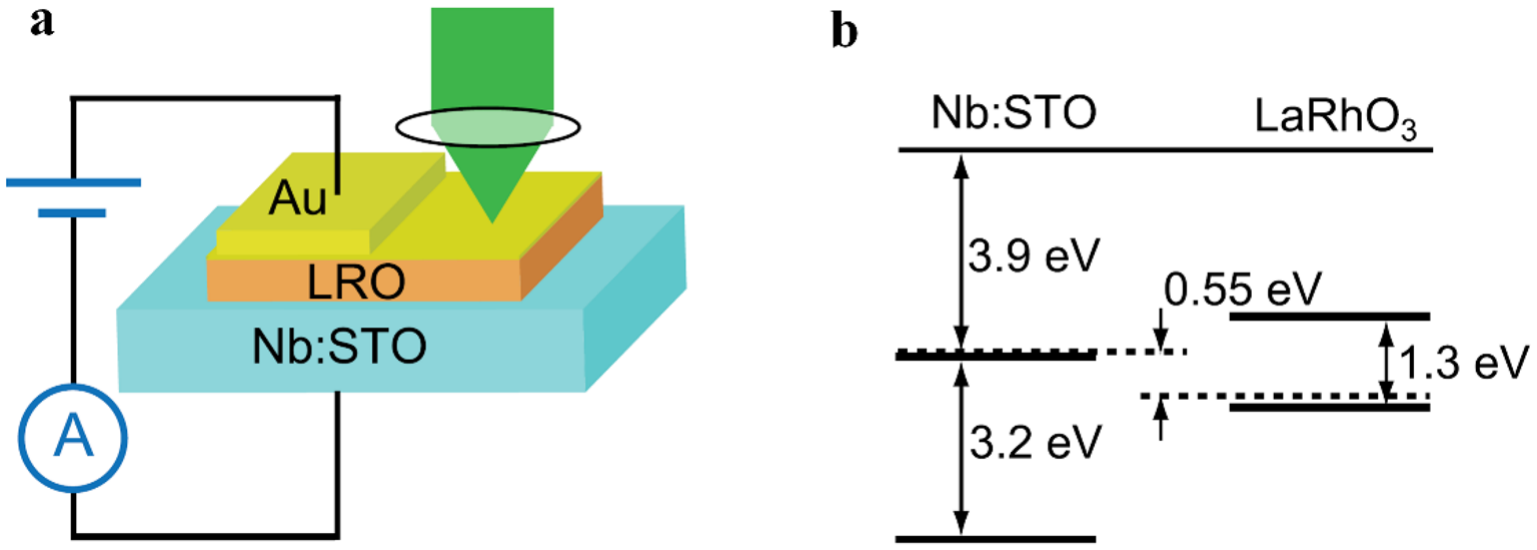
**a** Structure of p-LRO/n-Nb:STO heterojunction. **b** Band alignment relation of LRO and Nb:STO [135]

Due to their high chemical stability, ABO_3_-based perovskite transition metal oxides are of great interest in p-type materials. Even with A- or B-site doping, it is still stable for perovskite structures. Lanthanide-based perovskite LaRhO_3_ (LRO) with A-site doping (substituting La for Sr or Ca) was reported as a candidate for p-type semiconductors [134]. Nakamura et al. [135] reported a p-type single-crystalline LRO semiconductor and investigated the physical properties of a heterojunction, which was comprised of n-type Nb:SrTiO_3_. The bandgap of p-type LRO was just 1.3 eV, as shown Fig. 4.

In comparison with Cu^+^-based p-type oxides, the localization of O 2p orbitals is reduced from hybridization with metal d orbitals using a method called “chemical modulation of the valence band” (CMVB) [136]. The correlated metal oxides, Cr_2_O_3_, V_2_O_3_, LaCrO_3_, LaVO_3_, and LaCoO_3_, were identified to be p-type TCOs [33, 42, 137–139]. The VB consists of a hybridization between O 2p orbitals and transition metal 3d orbitals, leading to a reduction of the hole localization at the top of VB. In A-site doped perovskites LaBO_3_ (B = V, Cr, Co), the holes at the top of valence band (VB) could be introduced by replacing Sr^2+^ for La^3+^. Zhang et al. [42] grew a new p-type Sr-doped LaCrO_3_ on SrTiO_3_ substrate by molecular beam epitaxy. The conductivity was up to ~ 50 S cm^−1^ due to a high hole carrier concentration of 7.5 × 10^21^ cm^−3^. In the periodic table, V element is located to the left side of Cr. Therefore, the size of the 3d wavefunction of V^3+^ is expanded, resulting in a smaller U (d–d Coulomb repulsion energy) in LaVO_3_ than that of LaCrO_3_ [140]. The conductivity of p-type La_2/3_Sr_1/3_VO_3_ (LSVO) films was enhanced to nearly ~ 900 S·cm^−1^ at room temperature [33]. Meanwhile, the films exhibited a high transmittance up to 70%, indicating that they possess the potential to be a high performance p-type TCO. Takashima et al. [139] grew p-type La_0.67_Sr_0.33_CoO_3_ films on a SrTiO_3_ substrate by controlling the oxygen atmosphere and prepared p-n heterostructure. Furthermore, p-BaSnO_3_:K/n-BaSnO_3_:La whole perovskite p-n junctions were reported, and results show that they are stable at elevated temperatures (up to 300 °C) [141].

With regard to B-site doped materials, researchers substitute tetravalent ions on B-site by trivalent ions to prepare p-type perovskite oxides. SrTi_1-x_Sc_x_O_3_ is a p-type semiconductor with Sc^3+^ replacing Ti^4+^ in SrTiO_3_. However, the acceptor levels were so deep that the holes were localized [60]. Guo et al. [142] reported In-doped SrTiO_3_ films to create hole carriers and investigated the optical properties. The average transmittance is up to 80% in the visible region. For Al-doped SrSnO_3_ (Eg > 3.9 eV), the transmittance was increased, too [62].

Perovskites BaSnO_3_ and BaZrO_3_ doped by trivalent elements (Ga, Sc, In, Y) are able to incorporate oxygen at low temperatures. The hole concentration decreases at elevated temperatures [143]. Wang et al. reported that Y-doped BaSnO_3_ showed p-type behavior under a controlled atmosphere [144]. It is well known that the electronic conductivity and Seebeck Coefficient of semiconducting materials are measured by high-temperature sintering techniques. These procedures may lead to unreliable results such as changing transport properties from p- to n-type [145]. The holes induced by acceptor-doped perovskite oxides are compensated by intrinsic defects (oxygen vacancies) [146–148]. Acceptor-doped perovskite, BaSnO_3_, has also gained attention in various electrochemical devices [149, 150].

## Conclusion and outlook

In this article, we comprehensively review the recent developments of perovskites with both high carrier concentration and mobility. SrVO_3_ with strong electron–electron interactions exhibits a higher carrier concentration of 2.2 × 10^22^ cm^−3^ than that of ITO. It also maintains a high conductivity of 10^4^ S·cm^−1^ and optical transmittance in the visible range by taking advantage of its lower plasma frequency (ω_p_* = 1.33 eV). The performance of SrVO_3_ films are affected by crystallinity, mismatched substrate, and growth condition. A low resistivity of 28 μΩ·cm was achieved at room temperature in SVO films, which were grown by hybrid MBE in order to reduce the number of defects. Various lattice matched materials, including (LaAlO_3_)_0.3_(Sr_2_AlTaO_6_)_0.7_ (LSAT), SrTiO_3_ (STO), and NdGaO_3_ (NGO) have been used as substrates to prepare SVO films. Meanwhile, the performances of SVO thin films are found sensitive to oxygen pressure. Due to the high effective mass, the carrier mobility is just on the order of 10 cm^2^·V^−1^·s^−1^. On the other hand, BaSnO_3_-based perovskites with dispersive Sn 5 s states on the CBM, leading to a small carrier effective mass, have the highest carrier mobility of 320 cm^2^·V^−1^·s^−1^ in the single crystals and 183 cm^2^·V^−1^·s^−1^ in the epitaxial films at room temperature. It is easy to substitute Ba^2+^ by La^3+^ in BaSnO_3_ to introduce electron carriers due to the low formation energy of La_Ba_. Researchers have also managed to grow LBSO films on lattice matched substrates or insert a buffer layer between the LBSO and the substrate to enhance the carrier mobility. The performance of LBSO film is also affected by oxygen vacancy which could be controlled by depositional condition. Because of the high performance of SVO and LBSO films, they are expected to be excellent candidates of TCOs to further optimize the physical properties.

Moreover, perovskites with chemical and structural stability could be doped on A- or B-site by low valence metal ions to create stable holes in the valence band. Although various acceptor-doped perovskite oxides have been reported, their physical properties are still inferior to the corresponding n-type materials. It is necessary to further optimize the performance of p-type perovskites. Furthermore, perovskite oxides can be doped as both n- and p-type conductors, so full perovskite devices might be possible eventually.

## Data Availability

No.
